# Fibrosarcoma Originating from the Pleura in Cotton-Top Tamarin [*Oedipomidas* (*Saguinus*) *oedipus*, Linnaeus 1758]

**DOI:** 10.3390/ani16020195

**Published:** 2026-01-09

**Authors:** János Gál, Endre Sós, Árisz Ziszisz, Márton Hoitsy, Míra Mándoki, Dóra Csatári, Zoltán Vincze

**Affiliations:** 1 Department of Exotic Animal, Wildlife, Fish and Honeybee Medicine, University of Veterinary Medicine, István u. 2, 1078 Budapest, Hungary; gal.janos@univet.hu (J.G.); drsos.endre@zoobudapest.com (E.S.); ziszisz.arisz@univet.hu (Á.Z.); hoitsym@gmail.com (M.H.); csatari.dora@univet.hu (D.C.); 2 Budapest Zoo and Botanical Garden, Állatkerti Krt. 6-12, 1146 Budapest, Hungary; 3 Department of Pathology, University of Veterinary Medicine, 1078 Budapest, Hungary; mandoki.mira@univet.hu

**Keywords:** fibrosarcoma, Cotton-top tamarin, *Saguinus oedipus*, non metastatic, neoplasms

## Abstract

This report describes postmortem findings after the sudden death of a captive-bred middle-aged male cotton-top tamarin (*Saguinus oedipus*). A firm structure was attached to the pleura in the chest cavity and was assessed as a primary tumor without obvious metastasis. Histologically, the mass showed heterogenicity with immunohistochemistry (IHC) reactivity to vimentin, suggesting a diagnosis of fibrosarcoma of mesenchymal origin.

## Simple Summary

Different species of the subfamily Callitrichinae, including tamarins and marmosets, suffer and often succumb to malignant neoplastic diseases, with predominantly enteral symptoms, ranging from colitis or partial obstruction to small bowel diarrhea. The underlying primary neoplasia (carcinoma, adenocarcinoma) often has metastasis to diverse abdominal organs (lymph nodes, liver, pancreas). Other different tumors (adenoma, cystadenoma, fibroma, liposarcoma, fibrosarcoma) are also quite common in these New World primates.

## 1. Introduction

The cotton-top tamarin (*Saguinus oedipus*) belongs to the taxonomic order Primates and the family Cebidae. The species is listed on CITES Appendix I as a taxon threatened by extinction (with its relatives *S. bicolor*, *S. geoffroyi*, *S. leucopus*, *S. martinsi*) [1], so its trade is internationally regulated, permits are necessary for its captive care, and it is classified as Critically Endangered on the IUCN Red List. Accordingly, the taxon is a target species of the EEP (European Endangered Species Programme) initiated by EAZA (European Association of Zoo and Aquaria). Miranda Stevenson, based at Bristol Zoo Gardens, serves as the breeding coordinator for the cotton-top tamarin (*Saguinus oedipus*) [2]. The species is a small primate weighing 300 to 470 g inhabiting the northwestern forests of Colombia [3,4]. It searches for food (fruits, flowers, insects, small vertebrates, bird eggs) mainly in the arboreal zone, in pairs or small groups of 3–6 individuals [4]. After reaching sexual maturity at 18–24 months of age [5], adult females usually give birth to two offspring after a gestation period of 140 days. Their maximum lifespan in the wild is approximately 10 years [3].

Among marmosets and tamarins, the subfamily *Callitrichinae* is not only an endangered species, making it a target for breeding programs within zoos [2], but also a popular pet for hobbyists [6,7]. Recognized as having several biological and physiological similarities to humans, their small size, high fertility and ease of handling have resulted in common marmosets (*Callithrix jacchus*) being increasingly used in neuroscience and biomedical research, as NHPs, or Non-Human-Primates [8].

Among the species belonging to the family *Cebidae* (New World monkeys) and the subfamily *Callitrichinae* (marmosets), several neoplastic diseases have been reported. The incidence rate of chronic colitis was investigated in cotton-top tamarins (*Saguinus oedipus*). Based on biopsy samples collected by colonoscopy, colitis was present at an incidence rate of 64.5% among 69 captive monkeys. Carcinoma types were confirmed histologically in 17.3% of the samples [9]. Besides the cotton-top tamarin (*Saguinus oedipus*), colonic adenocarcinoma was also found in Goeldi’s marmoset (*Callimico goeldii*). The cancers originated from the mucosa and showed early invasion of the submucosa, lymphatic apparatus, and paracolonic lymph nodes [10]. Chronic colitis shows a strong association with invasive colonic adenocarcinoma with subsequent metastasis to colonic lymph nodes but not elsewhere, based on a study of 3.5 years following 210 cotton-top tamarins [11]. Similar results were demonstrated in a prospective study on cotton-top tamarins raised from birth through 5 years of age. Diet maily affected chronic mucosal changes in the colon, and changing environmental factors caused acute colitis; together, these led to colitis-associated colon cancer [12].

Mucinous mucoid adenocarcinoma was described in the colon of the same species, and in a close relative, the saddle-back tamarin (*Saguinus fuscicollis*), a well-differentiated, tubular mucoid adenocarcinoma was described at the ileocaecal valve, which also metastasized to the regional lymph nodes [13]. Mucinous adenocarcinoma was also found in an elderly, 9-year-old female cotton-top tamarin, metastasizing to the pancreas and regional mesenteric lymph nodes. This animal showed intermittent diarrhea prior to death [14]. Although colorectal carcinoma is quite common in the mentioned species, metastasis to the liver is less common [15,16].

Small intestinal carcinoma is one of the most common neoplastic causes of morbidity and mortality in older common marmosets. The average age of ten common marmosets diagnosed was 6.6 years with no sex predilection, affecting 90% of these in their proximal small intestine. Helicobacter-like bacteria were not found, nor was callitrichine herpesvirus detected in neoplastic intestinal sections [17].

Carcinomas may also occur in other organs. The first case of renal cell carcinoma (RCC) as a malignant form of urogenital cancer, arising from renal tubular epithelial cells, was very recently described in a female common marmoset (*C. jacchus*). Malignant renal tumors are quite often found in NHPs, with baboons showing a male sex predilection [18].

An aggressive hepatic fibrosarcoma was found after postmortem examination in a young adult *Callithrix jacchus*, and the diagnosis was confirmed by IHC [19].

An adult, 400 g, female common marmoset (*C. jacchus*) showed a firm swelling of the left forearm from wrist to elbow. The necropsy and IHC revealed a nerve sheath tumor (NST) originating from Schwann cells, a schwannoma, locally invasive, categorized based on the French grading system as grade II [20].

A 4-year-old male common marmoset (*C. jacchus*) was euthanized after exhibiting bilateral hind limb paralysis. Postmortem findings included an intramuscular mass in the lower right back, composed of spindle-shaped tumor cells. Immunohistochemically, the neoplastic cells showed positivity for myogenin, desmin, vimentin, and alpha-smooth muscle actin and the mass was diagnosed as a rhabdomyosarcoma [21].

A young, 9-month-old female common marmoset (*C. jacchus*) showed increased respiratory effort. Necropsy found an edematous abdominal subcutis, enlarged thymus, and 3 mL clear-red fluid in her thoracic cavity. Histology, including IHC, confirmed spontaneous mediastinal myeloid sarcoma [22].

Benign ovarian teratoma has been reported in a 3-year-old colony-bred common marmoset (*C. jacchus*) [23].

A liposarcoma showing myxofibrosarcoma-like features was reported in the abdominal cavity of a golden-headed lion tamarin (*Leontopithecus chrysomelas*). This case was diagnosed in a young specimen that died in the wild [24]. Two older females of this same species had hepatocellular carcinoma (HCC) reported, one of them with pulmonary metastasis. The IHC showed positivity for hepatocyte-positive antigen (HPA) [25].

In an elderly male cotton-top tamarin (*Saguinus oedipus*), a tumor originating from hormone-producing interstitial cells, resembling Sertoli cells in some areas of the testis while resembling granulosa cells in other areas, has been reported as well [26].

In Japan, a young common marmoset (*C. jacchus*) male was euthanized after a 4-month progression of predominantly gastrointestinal signs. The postmortem examination found lymphadenopathy. Multiple lymph nodes (submandibular, axillary, inguinal, and pericardial) showed asymmetrical enlargement. Additional lesions were noted in the thymus and spleen. Histopathology and IHC diagnosed malignant T cell lymphoma [27].

Tumorous lesions have also been described in the excretory system of species belonging to the New World monkey family (Cebidae). In a common marmoset (*C. jacchus*), a polycystic nephroblastoma was described in the kidney, without any metastasis evident to the visceral organs [28]. In another species, moustached tamarin (*Saguinus mystax*), haemangiosarcoma was observed in the renal cortex [29]. In the same species, an incidental cystadenoma was found in the lung on postmortem of a young individual that died after cranial trauma [30].

An endocrine tumor was reported in a 10-year-old male common marmoset (*C. jacchus*), which was diagnosed as a thyroid follicular adenoma with accumulation of collagen type IV [31]. A well-differentiated pulmonary adenocarcinoma was documented by ICH in a 7-year-old common marmoset (*C. jacchus*) female without any local or distant metastasis [32].

## 2. Materials and Methods

On 10 January 2025, the Department of Exotic Animal, Wildlife, Fish and Honeybee Medicine received the carcass of an approximately 8-year-old, 550 g male cotton-top tamarin (*Saguinus oedipus*) from a domestic zoo for diagnostic postmortem examination. The male tamarin died suddenly without showing any prior symptoms. It was kept with its mate in an enclosure measuring 2.5 × 6.5 m and 2.6 m high, where tree branches provided them with climbing opportunities. Wood chips were used as the substrate on the floor of the enclosure. In winter, the monkeys lived in an indoor enclosed habitat heated to support tropical temperatures. They were fed a mixture of chopped fruit, vegetables, boiled chicken eggs, and mealworms.

After external examination, the carcass was dissected lege artis. Samples of the organs showing lesions and the mass found in the chest cavity were fixed in 10% formaldehyde solution, then embedded in paraffin, sectioned into 3–4 µm thick slices, mounted on slides, and stained with hematoxylin and eosin. We performed IHC for SMA, desmin, cytokeratin, Ki-67, vimentin, and Factor VIII reactions on the thoracic mass using different antibodies (specifications in Table 1).

We performed bacterial culture in vitro from the lungs and chest cavity in a 37 °C thermostat under aerobic conditions for 24 h.

## 3. Results

**Figure 1:**
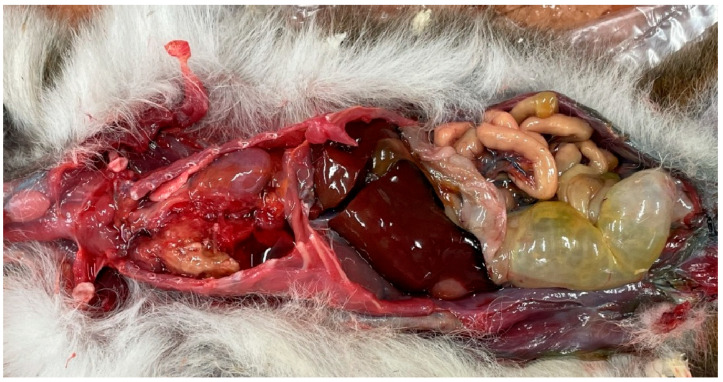
Cotton-top tamarin (*Saguinus oedipus*) with its chest cavity opened, showing the tissue growth pushing the heart aside.

The male tamarin was in good overall muscle, body, and nutritional condition. The fur and skin, as well as the natural body openings and the extremities, showed no pathological abnormalities. The subcutaneous connective tissue, skeletal muscles, and regional lymph nodes were also normal. Upon opening an appropriately shaped and firm abdominal cavity, we observed organs in their normal positions, with the peritoneal wall and visceral layers smooth, shiny, and translucent. The gastrointestinal tract showed only slight dilation of the jejunum with moderate gas accumulation (Figure 1).

**Figure 2:**
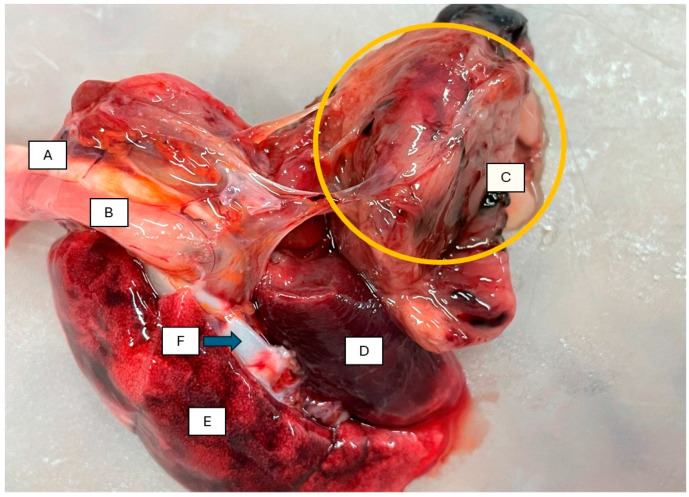
Tumor adherent to the pleura of the right lung (A. trachea, B. oesophagus, C. fibrosarcomatic mass, D. lung with atelectasis, E. lung with emphysema, F. aortic arch).

Abdominal viscera were normal including liver, spleen, and kidney. Approximately 5 mL of serosanguinous fluid was observed in the thoracic cavity. On the right side, an irregularly shaped, mottled tissue mass was visible, slightly displacing the pericardium and heart to the opposite side (Figure 1). The respiratory apparatus removed from the chest cavity was adherent to the pleura of the right lung by a dense, elastic, mottled, irregularly shaped, 45 × 32 × 28 mm tissue growth, which compressed the lung (Figure 2).

**Figure 3:**
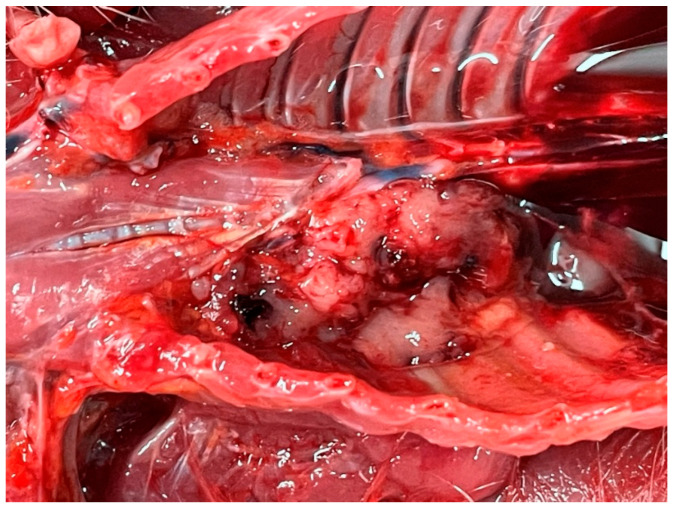
Fibrosarcoma closely attached to the parietal pleura.

This part of the lung was smaller, slightly denser to the touch, and uniform and dark red in color, with no crackle sound when incised, and yielded a moderate amount of watery content to the incision surface, containing no air bubbles, and with typical compressive atelectasis. The left lung was fluffy to the touch, normal in shape, and slightly enlarged, with a surface that was mottled with darker and lighter red areas. The incision site crackled when incised, a typical sign of emphysema, and a small amount of watery content containing many gas bubbles was expelled onto the incision surface. When the mediastinal organs were removed, part of the tumor was found to be closely attached to the anterior upper wall of the mediastinum near the spinal column (Figure 3).

The tumor was mottled, showing a nest-like structure. The pericardium, heart, and large airways showed no gross pathological abnormalities on portmortem. Aerobic bacterial cultures of the pleura fluid and the mass were negative.

**Figure 4:**
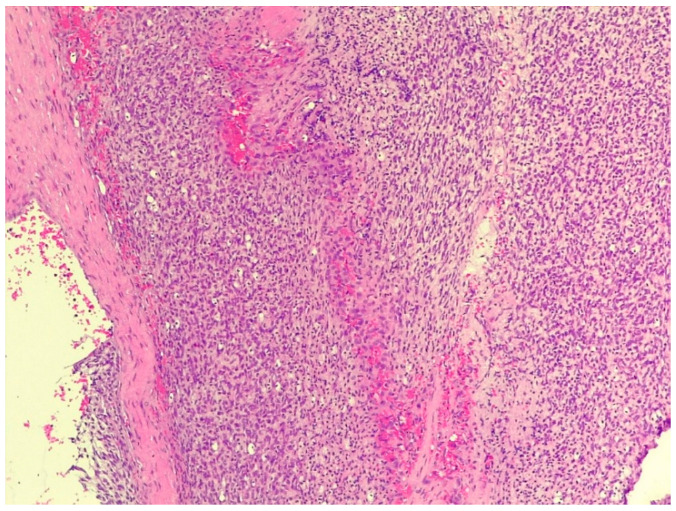
Irregularly arranged bundles composed of heterogeneous cell populations in fibrosarcoma (staining: H. E., M.: 120×).

In the histological sections taken from the thoracic tumor, a population of fusiform cells arranged in bundles with elongated to round nuclei with stippled chromatin was found. The tumor invaded the parietal pleura, whereas the intercostal muscles were preserved (Figure 4).

**Figure 5:**
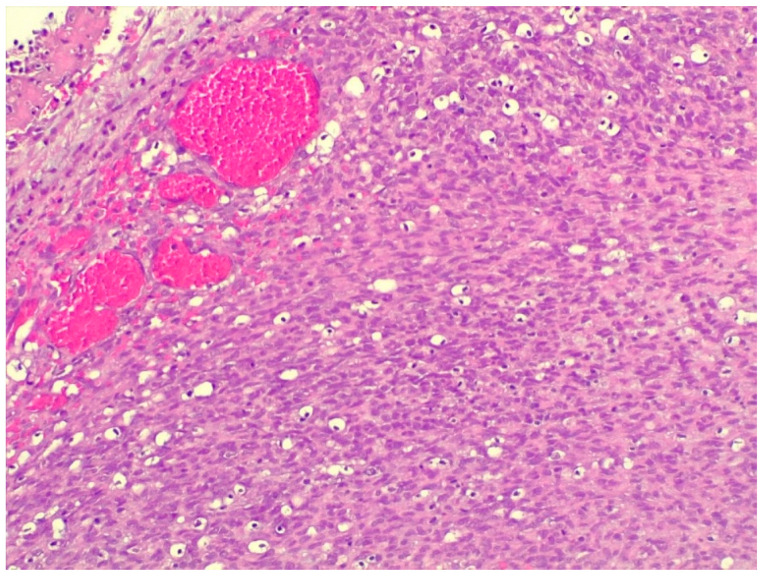
Clusters formed by immature, hyperchromatic cells; blood vessel cross-sections are also visible (staining: H. E., M.: 120×).

A more developed blood vessel network was observed among the cell populations of the tumor, which was patchy in structure in places and contained more cells, as well as more prominent, hyperchromatic, and rounder cell nuclei (Figure 5).

Here, the blood vessels showed varying widths and irregular shapes, but the larger vessels were filled with blood. The tumor was typed using human antibodies. In the vimentin (V9 clone, Agilent Dako, dilution 1:100) reaction performed on the section, we obtained homogeneous, intense positivity across the entire tumor area.

**Figure 6:**
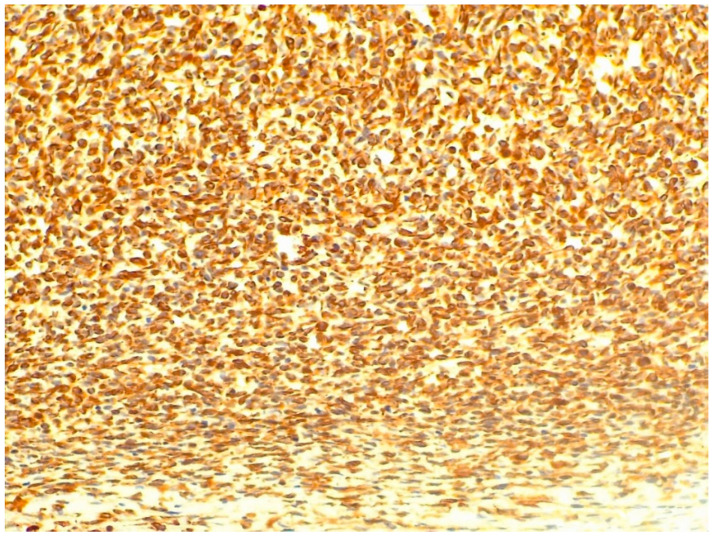
Intense positivity with vimentin immunohistochemical reaction in the higher cell-dense area of the tumor (staining: vimentin, M.: 120×).

This reaction confirmed the mesenchymal origin of the tumor and distinguished it from rhabdomyosarcomas and leiomyosarcomas, as positivity can be observed in non-muscle cell types (including lymphocytes, fibroblasts, and melanocytes) (Figure 6).

**Figure 7:**
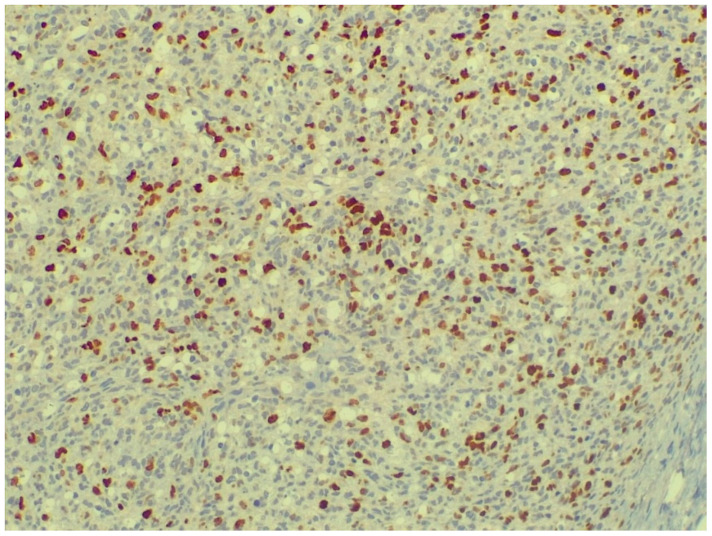
Ki67-positive, mitotic cell figures in the tumor (staining: Ki67, M.: 120×).

The fibrosarcoma we found could be distinguished from the former based on the morphology of the cells forming the tumor parenchyma. The Ki-67 reaction was clearly positive and allowed us to determine the proliferative index (Figure 7), which was 11.66. Although, based on recent studies [33], the Ki67 global digital score (Ki67-GDS) showed greater prognostic relevance than the mitotic count (MC), the fully automatic Ki67 hotspot digital score (Ki67-faHDS), and the semi-automatic Ki67 hotspot digital score (Ki67-saHDS), this study was conducted from a pathological perspective, where, unlike in clinical cases, prognosis did not play a role, so our study was based on Ki67-saHDS.

**Figure 8:**
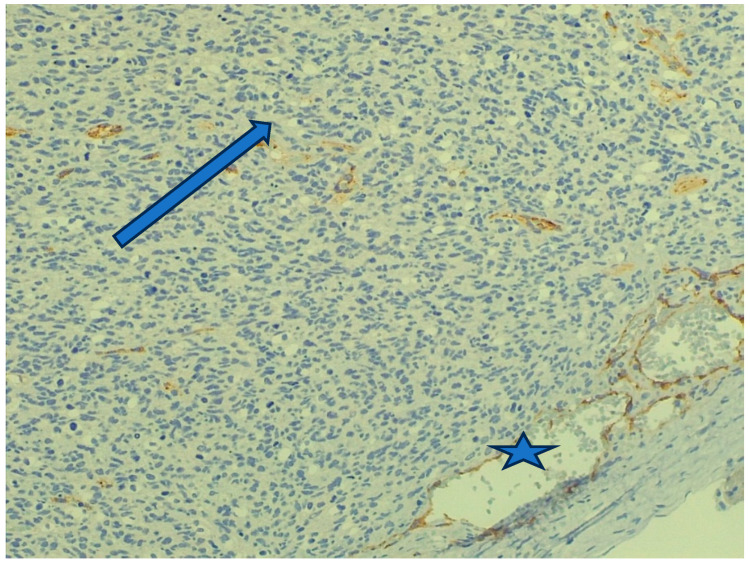
Capillaries detected with endothelial (Factor VIII) marker in the tumor parenchyma (arrow). Internal positive control is the larger vein running along the pleura (star). (Staining: Factor VIII, M.: 120×).

The vascularization of the tumor varied from area to area. There were parts of the tumor where it was very pronounced (Figure 8), but there were also poorly vascularized areas where more mature cell populations were arranged in bundles, forming the tumor parenchyma. The more developed blood supply was observed in areas of the tumor where hyperchromatic cells with more rounded nuclei were present and the area appeared more cell-dense. To demonstrate this, we used an endothelin (Factor VIII) reaction, causing the endothelial cells of the blood vessels to have an increased mahogany-brown color intensity.

**Figure 9:**
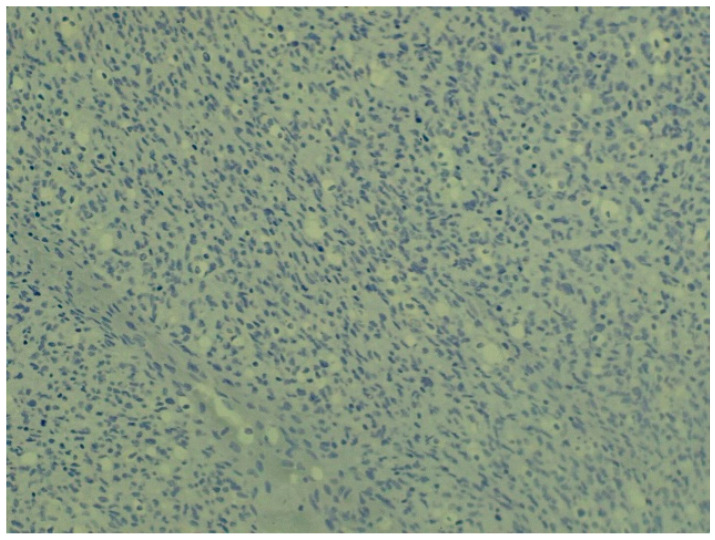
Negative reaction for desmin (D33 clone, Agilent Dako, dilution 1:75) (staining: desmin, M.: 120×).

**Figure 10:**
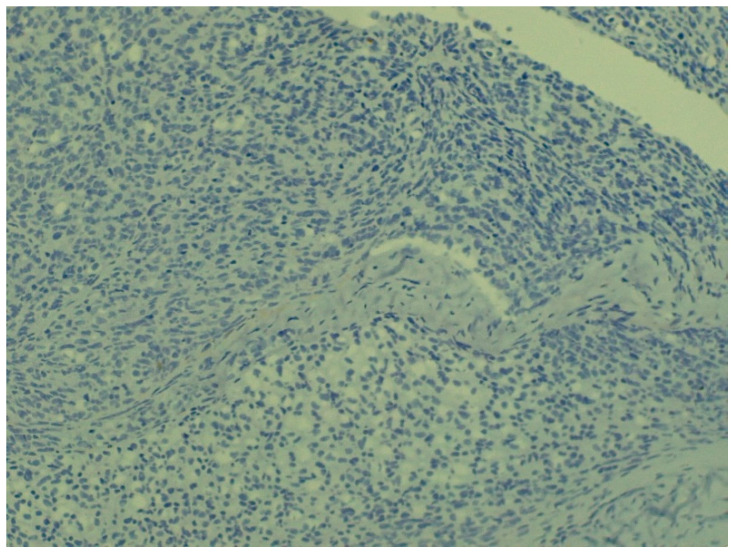
Negative reaction for smooth muscle actin—SMA (1A4 clone, Agilent Dako, dilution 1:75) (staining: SMA, M.: 120×).

**Figure 11:**
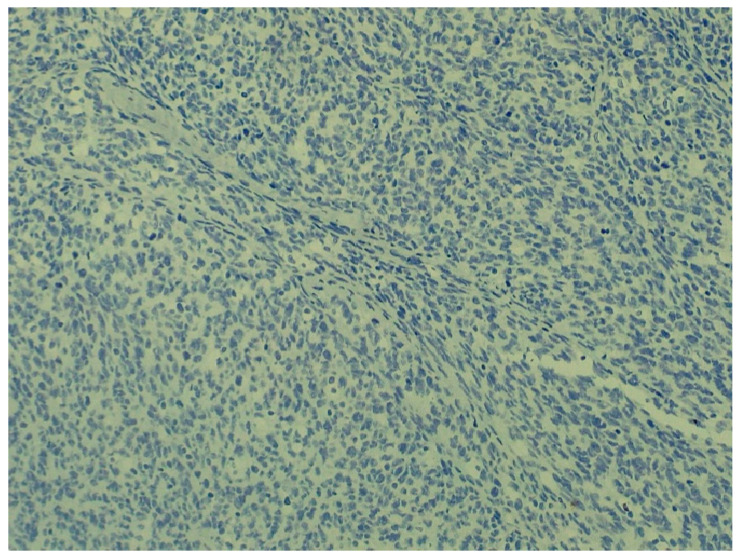
Negative reaction for pancytokeratin (AE1/AE3 clone, Agilent Dako, dilution 1:150) (staining: pancytokeratin, M.: 120×).

Further IHC tests were performed to rule out other tissue origins (Figure 9, Figure 10 and Figure 11) with desmin, SMA, and pancytokeratin markers, which all showed negative results.

## 4. Conclusions

This was the first case to describe a fibrosarcoma in a cotton-top tamarin (*Saguinus oedipus*) that distorted the thoracic cavity, originating from the pleural wall. The thoracic tumor compressed the lung in the affected area, was connected to its serous membrane, did not form metastases, and had a heterogeneous structure. The thoracic tumor had significant space-occupying pathology that impaired gas exchange in the body. Tumors of epithelial origin in the digestive tract, such as carcinoma, are not uncommon in cotton-top tamarins, but we have not found any data on primary mesenchymal tumors in the thoracic cavity in the available literature.

## Figures and Tables

**Table 1:** Antibody specification applied for IHC.

Name	Origin	Clone	Producer	Dilution
SMA	Monoclonal mouse, anti-human actin (smooth muscle) antibody	1A4	Agilent Dako (Santa Clara, CA, USA)	1:75
Desmin	Monoclonal mouse, anti-human desmin antibody	D33	Agilent Dako	1:75
Cytokeratin	Monoclonal mouse, anti-human cytokeratin antibody	AE1/AE3	Agilent Dako	1:150
Vimentin	Monoclonal mouse, anti-human vimentin antibody	V9	Agilent Dako	1:100
Factor VIII	Polyclonal antibody, anti-human	Von Willebrand Factor	Agilent Dako	1:450
Ki67	Monoclonal	MIB-1	Biocare Medical (Pacheco, CA, USA)	Ready-to-use

## Data Availability

The raw data supporting the conclusions of this article will be made available by the authors on request.
